# Levocetirizinium dipicrate

**DOI:** 10.1107/S1600536810045733

**Published:** 2010-11-13

**Authors:** Jerry P. Jasinski, Ray J. Butcher, M. S. Siddegowda, H. S. Yathirajan, A. R. Ramesha

**Affiliations:** a Department of Chemistry, Keene State College, 229 Main Street, Keene, NH 03435-2001, USA; b Department of Chemistry, Howard University, 525 College Street NW, Washington, DC 20059, USA; c Department of Studies in Chemistry, University of Mysore, Manasagangotri, Mysore 570 006, India; d RL Fine Chem., Bangalore 560 064, India

## Abstract

There are two cation–dianion pairs in the asymmetric unit of the title compound, C_21_H_27_ClN_2_O_3_^2+^·2C_6_H_2_N_3_O_7_^−^ {systematic name: 1-[2-(carb­oxy­meth­oxy)eth­yl]-4-[(*R*)-(4-chloro­phen­yl)phenyl­meth­yl]piperazine-1,4-diium bis­(2,4,6-trinitro­phenol­ate)}. The piperazine group in the levocetirizinium cation is protonated at both N atoms. The acetyl end groups form *R*_2_^2^(8) hydrogen-bonded motifs with adjacent cations. Each picrate anion inter­acts with the proponated N atom in the cation through a bifurcated N—H⋯O hydrogen bond, forming *R*_1_^2^(6) ring motifs. Strong and weak inter­molecular N—H⋯O and strong O—H⋯O hydrogen bonds, and weak π–ring and π–π stacking inter­actions [centroid–centroid distance = 3.7419 (14) Å] dominate the crystal packing, creating a three-dimensional supra­molecular structure.

## Related literature

For related background, see: Hair & Scott, (2006 ▶). For related structures, see: Jasinski *et al.* (2009 ▶, 2010*a* ▶,*b* ▶). For bond-length data, see: Allen *et al.* (1987 ▶).
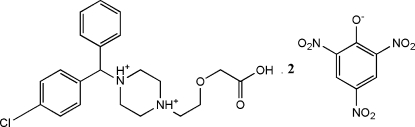


## Experimental

### 

#### Crystal data

C_21_H_27_ClN_2_O_3_^2+^·2C_6_H_2_N_3_O_7_^−^
*M* *_r_* = 847.11
Monoclinic, 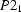

*a* = 11.2444 (1) Å
*b* = 15.7720 (2) Å
*c* = 20.6204 (2) Å
β = 95.998 (1)°
*V* = 3636.94 (7) Å^3^
*Z* = 4
Cu *K*α radiation
μ = 1.74 mm^−1^
*T* = 123 K
0.51 × 0.47 × 0.34 mm

#### Data collection

Oxford Diffraction Xcalibur Ruby Gemini diffractometer
Absorption correction: multi-scan (*CrysAlis RED*; Oxford Diffraction, 2007 ▶) *T*_min_ = 0.533, *T*_max_ = 1.000
14383 measured reflections
11120 independent reflections
10728 reflections with *I* > 2σ(*I*)
*R*_int_ = 0.021

#### Refinement

*R*[*F* ^2^ > 2σ(*F* ^2^)] = 0.044
*wR*(*F* ^2^) = 0.122
*S* = 1.03
11120 reflections
1063 parameters
1 restraint
H-atom parameters constrained
Δρ_max_ = 0.96 e Å^−3^
Δρ_min_ = −0.64 e Å^−3^
Absolute structure: Flack (1983 ▶), 3692 Friedel pairs
Flack parameter: 0.058 (13)

### 

Data collection: *CrysAlis PRO* (Oxford Diffraction, 2007 ▶); cell refinement: *CrysAlis PRO* data reduction: *CrysAlis RED* (Oxford Diffraction, 2007 ▶); program(s) used to solve structure: *SHELXS97* (Sheldrick, 2008 ▶); program(s) used to refine structure: *SHELXL97* (Sheldrick, 2008 ▶); molecular graphics: *SHELXTL* (Sheldrick, 2008 ▶); software used to prepare material for publication: *SHELXTL*.

## Supplementary Material

Crystal structure: contains datablocks global, I. DOI: 10.1107/S1600536810045733/om2375sup1.cif

Structure factors: contains datablocks I. DOI: 10.1107/S1600536810045733/om2375Isup2.hkl

Additional supplementary materials:  crystallographic information; 3D view; checkCIF report

## Figures and Tables

**Table 1:** Hydrogen-bond geometry (Å, °) *Cg*5 is the centroid of the C6*B*–C11*B* ring.

*D*—H⋯*A*	*D*—H	H⋯*A*	*D*⋯*A*	*D*—H⋯*A*
O2*A*—H2*AD*⋯O2*B*	0.84	1.80	2.638 (4)	180
N1*A*—H1*AC*⋯O1*D*	0.93	1.83	2.682 (3)	152
N1*A*—H1*AC*⋯O7*D*	0.93	2.63	3.301 (3)	129
N2*A*—H2*AC*⋯O1*C*	0.93	1.89	2.765 (3)	155
N2*A*—H2*AC*⋯O7*C*	0.93	2.46	2.990 (3)	116
O3*B*—H3*BC*⋯O3*A*	0.84	1.76	2.601 (4)	180
N1*B*—H1*BC*⋯O1*E*	0.93	1.85	2.678 (3)	147
N1*B*—H1*BC*⋯O7*E*	0.93	2.52	3.193 (3)	130
N2*B*—H2*BC*⋯O1*F*	0.93	1.91	2.764 (3)	153
N2*B*—H2*BC*⋯O7*F*	0.93	2.57	3.078 (4)	115
C19*B*—H19*C*⋯*Cg*5^i^	0.99	2.95	3.792 (4)	144

Symmetry code: (i) 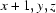.

**Fig. 1.**
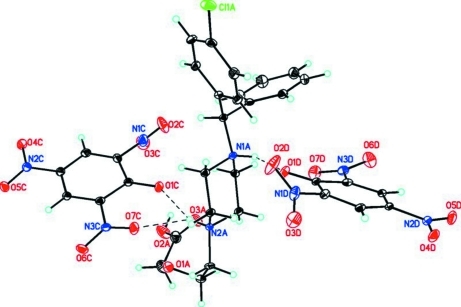
Molecular structure of the title compound showing the atom labeling scheme and 50% probability displacement ellipsoids. Dashed lines indicate strong and weak N—H···O intermolecular hydrogen bonds

**Fig. 2.**
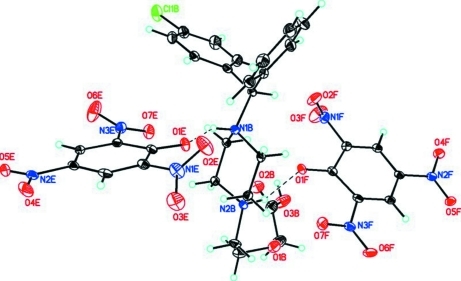
Molecular structure of the title compound showing the atom labeling scheme and 50% probability displacement ellipsoids. Dashed lines indicate strong and weak N—H···O intermolecular hydrogen bonds

**Fig. 3.**
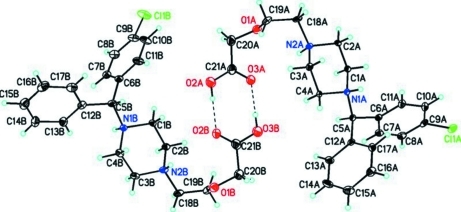
Molecular structure of adjacent cations from the title compound showing the acetyl end groups forming an R22(8) hydrogen bonded motif.

**Fig. 4.**
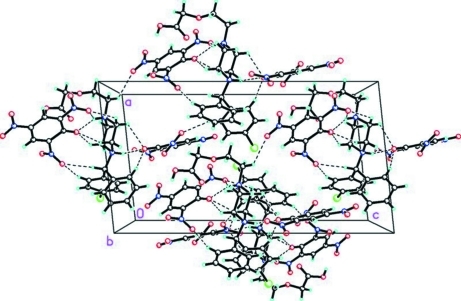
Packing diagram of the title compound viewed down the b axis. Dashed lines indicate strong and weak intermolecular N—H···O and O—H···O hydrogen bond interactions creating a 3-D supramulecular structure.

## supplementary crystallographic information

### Comment

Levocetirizine (as levocetirizine dihydrochloride) is a third-generation non-sedative antihistamine, developed from the second-generation antihistamine cetirizine.Chemically, levocetirizine is the active enantiomer of cetirizine. It is the *L*-enantiomer of the cetirizine racemate. Levocetirizine works by blocking histamine receptors. It does not prevent the actual release of histamine from mast cells, but prevents it from binding to its receptors. This in turn prevents the release of other allergy chemicals and increased blood supply to the area, and provides relief from the typical symptoms of hayfever. Levocetirizine is called a non-sedating antihistamine as it does not enter the brain in significant amounts, and is therefore unlikely to cause drowsiness. A review on the use of levocetirizine in the management of allergic rhinitis and skin allergies is described (Hair & Scott, 2006).

Recently, the crystal structures of propiverine picrate (Jasinski *et al.*, 2009), imatinibium dipicrate (Jasinski *et al.*, 2010*b*) and chlorimipraminium picrate (Jasinski *et al.*, 2010*a*) have been reported. The present work reports the crystal structure of the salt, C_21_H_27_ClN_2_O_3_^2+^. 2C_6_H_2_N_3_O_7_^-^, formed by the interaction between 2-[2-[4-[(*R*)-(4-chlorophenyl)-phenyl-methyl] piperazin-1-yl]ethoxy]acetic acid and 2,4,6-trinitrophenol in aqueous medium.

In the crystal structure of the title compound the 6-membered piperazine groups (N1A/C1A/C2A/N2A/C3A/C4A & N1B/C1B/C2B/N2B/C3B/C4B) in the levocetirizinium cation are protonated at both N atoms (Fig. 1) and adopt slightly distorted chair conformations with puckering parameters Q, θ and φ of 0.591 (3)A% & 0.583 (3) Å, 171.6 (3)° & 170.8 (3)°, and 353.0 (17)° & 358.2 (19)°, for molecules A & B respectively (Figs.1 & 2). For an ideal chair θ has a value of 0 or 180°. Bond distances (Allen *et al.*, 1987) and angles are in normal ranges . *R*_2_^1^(6) graph-set motifs are formed between piperazine N1A—H1AC and N2A—H2AC groups and the picrate anions labeled D and C (Fig. 1) and piperazine N1B—H1BC and N2B—H2BC groups and the picrate anions labeled E and F (Fig. 2) through bifurcated N—H···O hydrgen bonds (Table 1). The acetyl end groups form an *R*_2_^2^(8) hydrogen bonded motif with adjacent cations (Fig. 3). The dihedral angle between the mean planes of the anion benzene ring pairs is 31.9 (2)Å (C—D) and 37.9 (6)Å (E—F), respectively.

The mean plane of the two *o*-NO_2_ groups in the two picrate anions are twisted by 15.8 (6)°, 53.7 (3)Å (ring C),25.9 (9) Å, 38.5 (1)Å (ring D), 24.5 (0) Å, 38.7 (2)Å (ring E) and 10.3 (3) Å, 56.9 (9)Å (ring F) with respect to the mean planes of the 6-membered benzene rings. The *p*-NO_2_ groups in both picrate anions are nearly in the plane of the ring (torsion angles O4C/N2C/C4C/C3C = -8.8 (4)°; O4D/N2D/C4D/C3D = -175.8 (2)°; O4E/N2E/C4E/C3E = 2.6 (4)°; O4F/N2F/C4F/C3F = 3.4 (4)°;). An extensive array of strong and weak N—H···O and strong O—H···O intermolecular hydrogen bonds (Table 1), weak π-ring (Table 2) and π-π (Table 3) stacking interactions dominate crystal packing in the unit cell creating a 3-D supramulecular structure (Fig. 4).

### Experimental

Levocetirizine (3.89 g, 0.01 mol) was dissolved in 20 ml of methanol and picric acid (2.4 g, 0.01 mol) was dissolved in 20 ml of methanol. Both the solutions were mixed and stirred in a beaker at room temperature for 1/2 half hour. The mixture was warmed for 10 min at 323 K & kept aside for two days at room temperature. The formed salt was filtered & dried in a vaccum desiccator over phosphorous pentoxide. The salt was recrystallized from dimethylsulphoxide by slow evaporation (m.p: 454–456 K).

### Refinement

All of the H atoms were placed in their calculated positions and then refined using the riding model with Atom—H lengths of 1.00, 0.95Å (CH), 0.99Å (CH_2_), 0.93Å (NH), or 0.84Å (OH). Isotropic displacement parameters for these atoms were set to 1.2 times (NH), 1.2 (CH, CH_2_) or 1.5 (OH) times *U*_eq_ of the parent atom.

### Crystal data

**Table:**

C_21_H_27_ClN_2_O_3_^2+^·2C_6_H_2_N_3_O_7_^−^	*F*(000) = 1752
*M**_r_* = 847.11	*D*_x_ = 1.547 Mg m^−^^3^
Monoclinic, *P*2_1_	Cu *K*α radiation, λ = 1.54178 Å
Hall symbol: P 2yb	Cell parameters from 12871 reflections
*a* = 11.2444 (1) Å	θ = 4.7–73.9°
*b* = 15.7720 (2) Å	µ = 1.74 mm^−^^1^
*c* = 20.6204 (2) Å	*T* = 123 K
β = 95.998 (1)°	Block, yellow
*V* = 3636.94 (7) Å^3^	0.51 × 0.47 × 0.34 mm
*Z* = 4	

### Data collection

**Table:**

Oxford Diffraction Xcalibur Ruby Gemini diffractometer	11120 independent reflections
Radiation source: Enhance (Cu) X-ray Source	10728 reflections with *I* > 2σ(*I*)
graphite	*R*_int_ = 0.021
Detector resolution: 10.5081 pixels mm^-1^	θ_max_ = 74.1°, θ_min_ = 4.7°
ω scans	*h* = −9→13
Absorption correction: multi-scan (*CrysAlis RED*; Oxford Diffraction, 2007)	*k* = −19→18
*T*_min_ = 0.533, *T*_max_ = 1.000	*l* = −25→24
14383 measured reflections	

### Refinement

**Table:**

Refinement on *F*^2^	Secondary atom site location: difference Fourier map
Least-squares matrix: full	Hydrogen site location: inferred from neighbouring sites
*R*[*F*^2^ > 2σ(*F*^2^)] = 0.044	H-atom parameters constrained
*wR*(*F*^2^) = 0.122	*w* = 1/[σ^2^(*F*_o_^2^) + (0.0826*P*)^2^ + 1.9759*P*] where *P* = (*F*_o_^2^ + 2*F*_c_^2^)/3
*S* = 1.03	(Δ/σ)_max_ < 0.001
11120 reflections	Δρ_max_ = 0.96 e Å^−^^3^
1063 parameters	Δρ_min_ = −0.64 e Å^−^^3^
1 restraint	Absolute structure: Flack (1983), 3460 Friedel pairs
Primary atom site location: structure-invariant direct methods	Flack parameter: 0.058 (13)

### Special details

**Table:**

Geometry. All e.s.d.'s (except the e.s.d. in the dihedral angle between two l.s. planes) are estimated using the full covariance matrix. The cell e.s.d.'s are taken into account individually in the estimation of e.s.d.'s in distances, angles and torsion angles; correlations between e.s.d.'s in cell parameters are only used when they are defined by crystal symmetry. An approximate (isotropic) treatment of cell e.s.d.'s is used for estimating e.s.d.'s involving l.s. planes.
Refinement. Refinement of *F*^2^ against ALL reflections. The weighted *R*-factor *wR* and goodness of fit *S* are based on *F*^2^, conventional *R*-factors *R* are based on *F*, with *F* set to zero for negative *F*^2^. The threshold expression of *F*^2^ > σ(*F*^2^) is used only for calculating *R*-factors(gt) *etc*. and is not relevant to the choice of reflections for refinement. *R*-factors based on *F*^2^ are statistically about twice as large as those based on *F*, and *R*- factors based on ALL data will be even larger.

### Fractional atomic coordinates and isotropic or equivalent isotropic displacement parameters (Å^2^)

**Table:**

	*x*	*y*	*z*	*U*_iso_*/*U*_eq_	
Cl1A	1.43159 (6)	−0.07506 (5)	0.46063 (4)	0.03707 (19)	
O1A	0.51357 (19)	0.14380 (15)	0.62287 (11)	0.0322 (5)	
O2A	0.5911 (3)	0.2584 (2)	0.76889 (13)	0.0535 (7)	
H2AD	0.6533	0.2854	0.7821	0.080*	
O3A	0.6722 (2)	0.26091 (17)	0.67498 (11)	0.0385 (6)	
N1A	0.95454 (18)	0.17016 (15)	0.53050 (10)	0.0155 (4)	
H1AC	0.9645	0.1922	0.4896	0.019*	
N2A	0.69680 (19)	0.15302 (15)	0.53729 (11)	0.0174 (4)	
H2AC	0.7070	0.1310	0.5793	0.021*	
C1A	0.8903 (2)	0.08603 (17)	0.52236 (12)	0.0178 (5)	
H1AA	0.8923	0.0577	0.5653	0.021*	
H1AB	0.9322	0.0491	0.4933	0.021*	
C2A	0.7617 (2)	0.09740 (18)	0.49397 (12)	0.0181 (5)	
H2AA	0.7593	0.1232	0.4501	0.022*	
H2AB	0.7218	0.0414	0.4894	0.022*	
C3A	0.7555 (2)	0.23808 (18)	0.53789 (13)	0.0194 (5)	
H3AA	0.7120	0.2781	0.5639	0.023*	
H3AB	0.7521	0.2602	0.4928	0.023*	
C4A	0.8845 (2)	0.23205 (17)	0.56680 (12)	0.0162 (5)	
H4AA	0.9221	0.2887	0.5658	0.019*	
H4AB	0.8872	0.2142	0.6130	0.019*	
C5A	1.0771 (2)	0.15712 (18)	0.56848 (12)	0.0170 (5)	
H5AA	1.0633	0.1349	0.6125	0.020*	
C6A	1.1552 (2)	0.09331 (18)	0.53805 (13)	0.0187 (5)	
C7A	1.2402 (2)	0.05136 (19)	0.58085 (14)	0.0236 (6)	
H7AA	1.2406	0.0593	0.6265	0.028*	
C8A	1.3239 (3)	−0.00169 (19)	0.55710 (15)	0.0267 (6)	
H8AA	1.3825	−0.0295	0.5861	0.032*	
C9A	1.3206 (2)	−0.01337 (18)	0.49055 (16)	0.0251 (6)	
C10A	1.2349 (3)	0.0241 (2)	0.44723 (15)	0.0260 (6)	
H10A	1.2321	0.0128	0.4018	0.031*	
C11A	1.1524 (3)	0.07878 (19)	0.47100 (14)	0.0240 (6)	
H11A	1.0941	0.1063	0.4416	0.029*	
C12A	1.1408 (2)	0.24216 (18)	0.57884 (13)	0.0196 (5)	
C13A	1.1565 (3)	0.2778 (2)	0.64133 (14)	0.0291 (7)	
H13A	1.1260	0.2496	0.6768	0.035*	
C14A	1.2168 (3)	0.3543 (3)	0.65159 (16)	0.0390 (8)	
H14A	1.2260	0.3788	0.6939	0.047*	
C15A	1.2637 (3)	0.3954 (2)	0.60031 (17)	0.0366 (8)	
H15A	1.3063	0.4471	0.6075	0.044*	
C16A	1.2474 (3)	0.3596 (2)	0.53822 (16)	0.0308 (7)	
H16A	1.2787	0.3875	0.5029	0.037*	
C17A	1.1863 (3)	0.28368 (19)	0.52730 (14)	0.0223 (6)	
H17A	1.1754	0.2601	0.4847	0.027*	
C18A	0.5646 (2)	0.1559 (2)	0.51504 (14)	0.0260 (6)	
H18A	0.5514	0.1894	0.4743	0.031*	
H18B	0.5353	0.0976	0.5053	0.031*	
C19A	0.4949 (3)	0.1943 (2)	0.56542 (16)	0.0302 (7)	
H19A	0.5221	0.2531	0.5749	0.036*	
H19B	0.4088	0.1957	0.5494	0.036*	
C20A	0.4897 (3)	0.1860 (3)	0.68060 (18)	0.0403 (8)	
H20A	0.4714	0.1436	0.7135	0.048*	
H20B	0.4187	0.2228	0.6712	0.048*	
C21A	0.5937 (3)	0.2386 (2)	0.70758 (17)	0.0370 (8)	
Cl1B	0.18104 (10)	0.10273 (6)	0.86966 (5)	0.0527 (3)	
O1B	0.9688 (2)	0.43787 (17)	0.86713 (11)	0.0376 (5)	
O2B	0.7870 (2)	0.34294 (17)	0.80996 (12)	0.0391 (6)	
O3B	0.8678 (3)	0.3414 (2)	0.71568 (13)	0.0575 (8)	
H3BC	0.8046	0.3154	0.7025	0.086*	
N1B	0.53581 (19)	0.41964 (16)	0.97035 (10)	0.0190 (4)	
H1BC	0.5269	0.3949	1.0105	0.023*	
N2B	0.7932 (2)	0.42637 (17)	0.95643 (11)	0.0224 (5)	
H2BC	0.7814	0.4518	0.9156	0.027*	
C1B	0.5976 (2)	0.35768 (18)	0.92903 (12)	0.0198 (5)	
H1BA	0.5926	0.3792	0.8837	0.024*	
H1BB	0.5549	0.3027	0.9282	0.024*	
C2B	0.7275 (3)	0.34358 (19)	0.95376 (13)	0.0224 (6)	
H2BA	0.7332	0.3180	0.9978	0.027*	
H2BB	0.7642	0.3039	0.9244	0.027*	
C3B	0.7382 (3)	0.4805 (2)	1.00382 (14)	0.0250 (6)	
H3BA	0.7831	0.5344	1.0096	0.030*	
H3BB	0.7433	0.4515	1.0466	0.030*	
C4B	0.6090 (3)	0.49921 (18)	0.98106 (14)	0.0223 (6)	
H4BA	0.5748	0.5349	1.0140	0.027*	
H4BB	0.6047	0.5317	0.9398	0.027*	
C5B	0.4118 (2)	0.43970 (19)	0.93531 (13)	0.0221 (6)	
H5BA	0.4251	0.4663	0.8927	0.026*	
C6B	0.3450 (2)	0.3572 (2)	0.91958 (13)	0.0233 (6)	
C7B	0.3046 (3)	0.3084 (2)	0.96865 (15)	0.0263 (6)	
H7BA	0.3131	0.3283	1.0124	0.032*	
C8B	0.2515 (3)	0.2303 (2)	0.95391 (17)	0.0339 (7)	
H8BA	0.2244	0.1961	0.9873	0.041*	
C9B	0.2388 (3)	0.2028 (2)	0.88932 (17)	0.0365 (8)	
C10B	0.2738 (4)	0.2517 (3)	0.84000 (16)	0.0408 (8)	
H10B	0.2618	0.2328	0.7961	0.049*	
C11B	0.3272 (3)	0.3296 (2)	0.85527 (16)	0.0343 (7)	
H11B	0.3517	0.3643	0.8215	0.041*	
C12B	0.3390 (3)	0.50213 (19)	0.97057 (15)	0.0244 (6)	
C13B	0.3556 (3)	0.5196 (2)	1.03748 (15)	0.0292 (7)	
H13B	0.4170	0.4916	1.0645	0.035*	
C14B	0.2819 (3)	0.5781 (2)	1.06445 (18)	0.0353 (7)	
H14B	0.2957	0.5916	1.1095	0.042*	
C15B	0.1889 (3)	0.6167 (2)	1.0263 (2)	0.0417 (8)	
H15B	0.1369	0.6548	1.0452	0.050*	
C16B	0.1725 (3)	0.5990 (2)	0.9599 (2)	0.0427 (9)	
H16B	0.1091	0.6255	0.9332	0.051*	
C17B	0.2473 (3)	0.5434 (2)	0.93261 (17)	0.0338 (7)	
H17B	0.2361	0.5331	0.8870	0.041*	
C18B	0.9256 (3)	0.4166 (2)	0.97499 (15)	0.0327 (7)	
H18C	0.9391	0.3790	1.0135	0.039*	
H18D	0.9607	0.4727	0.9871	0.039*	
C19B	0.9876 (3)	0.3799 (3)	0.91983 (19)	0.0396 (8)	
H19C	1.0741	0.3730	0.9333	0.047*	
H19D	0.9535	0.3238	0.9070	0.047*	
C20B	0.9815 (4)	0.4014 (3)	0.80583 (19)	0.0483 (10)	
H20C	1.0471	0.3593	0.8105	0.058*	
H20D	1.0034	0.4461	0.7755	0.058*	
C21B	0.8681 (4)	0.3587 (3)	0.77753 (17)	0.0415 (8)	
O1C	0.79680 (19)	0.09712 (15)	0.65794 (10)	0.0274 (4)	
O2C	1.0086 (3)	0.1450 (2)	0.73266 (12)	0.0616 (10)	
O3C	0.9234 (3)	0.17726 (19)	0.81843 (16)	0.0577 (8)	
O4C	0.8609 (2)	−0.11502 (16)	0.91389 (10)	0.0331 (5)	
O5C	0.7121 (2)	−0.18315 (15)	0.86224 (11)	0.0313 (5)	
O6C	0.56936 (19)	−0.10817 (17)	0.64531 (11)	0.0361 (6)	
O7C	0.6664 (2)	−0.02135 (15)	0.59006 (10)	0.0309 (5)	
N1C	0.9382 (3)	0.1313 (2)	0.77214 (14)	0.0401 (7)	
N2C	0.7885 (2)	−0.12627 (17)	0.86580 (12)	0.0255 (5)	
N3C	0.6489 (2)	−0.05485 (17)	0.64152 (11)	0.0224 (5)	
C1C	0.7961 (2)	0.04300 (19)	0.70205 (13)	0.0205 (5)	
C2C	0.8661 (3)	0.0537 (2)	0.76457 (14)	0.0254 (6)	
C3C	0.8660 (3)	0.0012 (2)	0.81686 (14)	0.0249 (6)	
H3CA	0.9145	0.0127	0.8564	0.030*	
C4C	0.7922 (2)	−0.0702 (2)	0.81035 (13)	0.0223 (6)	
C5C	0.7203 (2)	−0.08658 (19)	0.75341 (13)	0.0212 (5)	
H5CA	0.6686	−0.1344	0.7505	0.025*	
C6C	0.7240 (2)	−0.03285 (19)	0.70048 (13)	0.0207 (6)	
O1D	0.93928 (19)	0.18889 (14)	0.40063 (9)	0.0249 (4)	
O2D	0.9747 (3)	0.05081 (17)	0.32418 (14)	0.0548 (8)	
O3D	0.8281 (2)	0.05186 (16)	0.24704 (12)	0.0424 (6)	
O4D	0.8374 (2)	0.30033 (16)	0.11042 (10)	0.0309 (5)	
O5D	0.8824 (2)	0.42219 (16)	0.15462 (11)	0.0380 (5)	
O6D	1.0061 (3)	0.44020 (16)	0.38295 (13)	0.0443 (6)	
O7D	0.9339 (2)	0.34861 (17)	0.44645 (10)	0.0364 (5)	
N1D	0.8989 (3)	0.08785 (16)	0.28649 (12)	0.0285 (6)	
N2D	0.8686 (2)	0.34472 (17)	0.15838 (11)	0.0231 (5)	
N3D	0.9598 (2)	0.37156 (16)	0.39307 (12)	0.0241 (5)	
C1D	0.9264 (2)	0.22591 (18)	0.34697 (12)	0.0160 (5)	
C2D	0.9020 (2)	0.18040 (18)	0.28569 (13)	0.0198 (5)	
C3D	0.8806 (2)	0.21806 (19)	0.22587 (12)	0.0179 (5)	
H3DA	0.8595	0.1850	0.1879	0.022*	
C4D	0.8903 (2)	0.30535 (18)	0.22165 (13)	0.0180 (5)	
C5D	0.9179 (2)	0.35465 (18)	0.27694 (13)	0.0186 (5)	
H5DA	0.9264	0.4143	0.2733	0.022*	
C6D	0.9327 (2)	0.31620 (18)	0.33673 (13)	0.0184 (5)	
O1E	0.5620 (2)	0.39892 (13)	1.09998 (9)	0.0253 (4)	
O2E	0.5266 (3)	0.53058 (18)	1.18380 (13)	0.0507 (7)	
O3E	0.6761 (3)	0.52363 (18)	1.25888 (13)	0.0496 (7)	
O4E	0.6578 (2)	0.2660 (2)	1.38424 (11)	0.0452 (7)	
O5E	0.6351 (2)	0.14505 (19)	1.33419 (12)	0.0447 (7)	
O6E	0.4915 (4)	0.14663 (19)	1.10720 (15)	0.0659 (10)	
O7E	0.5555 (2)	0.24316 (15)	1.04664 (10)	0.0331 (5)	
N1E	0.6011 (3)	0.49029 (19)	1.21902 (13)	0.0342 (6)	
N2E	0.6358 (2)	0.2233 (2)	1.33420 (13)	0.0336 (7)	
N3E	0.5367 (2)	0.21628 (17)	1.09969 (13)	0.0277 (5)	
C1E	0.5733 (2)	0.35792 (19)	1.15211 (13)	0.0194 (5)	
C2E	0.5990 (2)	0.39845 (19)	1.21537 (14)	0.0218 (6)	
C3E	0.6207 (2)	0.3559 (2)	1.27318 (13)	0.0248 (6)	
H3EA	0.6411	0.3861	1.3127	0.030*	
C4E	0.6126 (2)	0.2685 (2)	1.27344 (14)	0.0259 (6)	
C5E	0.5844 (2)	0.2231 (2)	1.21629 (14)	0.0239 (6)	
H5EA	0.5775	0.1631	1.2172	0.029*	
C6E	0.5666 (2)	0.26696 (19)	1.15802 (13)	0.0195 (5)	
O1F	0.68330 (18)	0.50280 (14)	0.84530 (10)	0.0261 (4)	
O2F	0.4523 (2)	0.4860 (2)	0.77442 (12)	0.0477 (7)	
O3F	0.5271 (3)	0.4344 (2)	0.69206 (16)	0.0716 (10)	
O4F	0.6482 (2)	0.71341 (16)	0.58982 (10)	0.0324 (5)	
O5F	0.79813 (19)	0.77792 (16)	0.64310 (10)	0.0305 (5)	
O6F	0.92948 (19)	0.69978 (19)	0.86099 (11)	0.0389 (6)	
O7F	0.8319 (2)	0.61047 (17)	0.91374 (10)	0.0344 (5)	
N1F	0.5248 (2)	0.48557 (18)	0.73525 (12)	0.0302 (6)	
N2F	0.7192 (2)	0.72331 (17)	0.63870 (11)	0.0230 (5)	
N3F	0.8502 (2)	0.64698 (18)	0.86315 (11)	0.0251 (5)	
C1F	0.6912 (2)	0.55608 (19)	0.80234 (13)	0.0201 (5)	
C2F	0.6167 (2)	0.55174 (19)	0.74045 (13)	0.0219 (6)	
C3F	0.6259 (2)	0.6021 (2)	0.68762 (13)	0.0210 (5)	
H3FA	0.5766	0.5930	0.6480	0.025*	
C4F	0.7102 (2)	0.66759 (19)	0.69346 (13)	0.0204 (5)	
C5F	0.7830 (2)	0.6799 (2)	0.75121 (13)	0.0219 (6)	
H5FA	0.8398	0.7246	0.7546	0.026*	
C6F	0.7730 (2)	0.6275 (2)	0.80350 (13)	0.0220 (6)	

### Atomic displacement parameters (Å^2^)

**Table:**

	*U*^11^	*U*^22^	*U*^33^	*U*^12^	*U*^13^	*U*^23^
Cl1A	0.0230 (3)	0.0272 (3)	0.0621 (5)	0.0041 (3)	0.0098 (3)	−0.0105 (3)
O1A	0.0289 (11)	0.0372 (12)	0.0308 (11)	−0.0023 (9)	0.0039 (9)	0.0049 (10)
O2A	0.0541 (16)	0.069 (2)	0.0402 (14)	−0.0227 (15)	0.0193 (12)	−0.0114 (14)
O3A	0.0374 (13)	0.0474 (14)	0.0326 (12)	−0.0101 (11)	0.0119 (10)	−0.0043 (11)
N1A	0.0137 (9)	0.0221 (11)	0.0104 (9)	0.0022 (9)	0.0003 (8)	0.0029 (8)
N2A	0.0118 (9)	0.0228 (11)	0.0170 (10)	−0.0004 (8)	−0.0011 (8)	0.0052 (9)
C1A	0.0172 (12)	0.0205 (13)	0.0155 (11)	0.0013 (10)	0.0005 (9)	0.0004 (10)
C2A	0.0178 (12)	0.0230 (13)	0.0124 (11)	0.0008 (10)	−0.0029 (9)	0.0004 (10)
C3A	0.0174 (12)	0.0214 (14)	0.0196 (12)	0.0036 (10)	0.0023 (10)	0.0030 (11)
C4A	0.0138 (11)	0.0211 (13)	0.0140 (11)	0.0000 (10)	0.0030 (9)	−0.0025 (10)
C5A	0.0150 (11)	0.0236 (13)	0.0122 (11)	0.0026 (10)	0.0003 (9)	0.0030 (10)
C6A	0.0138 (11)	0.0215 (13)	0.0204 (12)	0.0013 (10)	0.0001 (10)	0.0044 (11)
C7A	0.0225 (13)	0.0262 (14)	0.0206 (13)	−0.0008 (11)	−0.0046 (10)	0.0059 (11)
C8A	0.0188 (13)	0.0245 (15)	0.0352 (16)	0.0037 (11)	−0.0048 (11)	0.0078 (13)
C9A	0.0147 (12)	0.0172 (13)	0.0445 (17)	0.0031 (10)	0.0091 (11)	−0.0003 (12)
C10A	0.0275 (14)	0.0285 (15)	0.0230 (14)	0.0031 (12)	0.0078 (11)	0.0018 (12)
C11A	0.0214 (13)	0.0271 (15)	0.0235 (13)	0.0081 (11)	0.0027 (11)	0.0065 (12)
C12A	0.0122 (11)	0.0267 (14)	0.0194 (12)	0.0003 (10)	−0.0008 (9)	0.0016 (11)
C13A	0.0307 (15)	0.0394 (18)	0.0164 (13)	−0.0086 (13)	−0.0012 (11)	0.0021 (12)
C14A	0.047 (2)	0.043 (2)	0.0244 (15)	−0.0132 (16)	−0.0075 (13)	−0.0055 (15)
C15A	0.0389 (18)	0.0336 (17)	0.0357 (17)	−0.0098 (14)	−0.0045 (14)	0.0048 (14)
C16A	0.0283 (15)	0.0328 (17)	0.0316 (15)	−0.0017 (13)	0.0046 (12)	0.0107 (14)
C17A	0.0217 (13)	0.0237 (14)	0.0215 (13)	0.0043 (11)	0.0019 (10)	0.0002 (11)
C18A	0.0127 (12)	0.0367 (16)	0.0267 (14)	−0.0022 (11)	−0.0072 (10)	0.0072 (13)
C19A	0.0143 (12)	0.0363 (17)	0.0397 (17)	0.0017 (11)	0.0021 (11)	0.0104 (14)
C20A	0.0407 (18)	0.045 (2)	0.0373 (18)	−0.0047 (16)	0.0161 (15)	−0.0020 (16)
C21A	0.0402 (18)	0.0380 (19)	0.0339 (17)	0.0000 (15)	0.0097 (14)	0.0020 (15)
Cl1B	0.0762 (7)	0.0329 (4)	0.0430 (5)	−0.0139 (4)	−0.0218 (4)	0.0062 (4)
O1B	0.0362 (12)	0.0415 (14)	0.0356 (12)	−0.0070 (10)	0.0058 (10)	−0.0004 (11)
O2B	0.0378 (12)	0.0464 (14)	0.0349 (12)	−0.0041 (11)	0.0125 (10)	−0.0074 (11)
O3B	0.0541 (16)	0.081 (2)	0.0408 (14)	−0.0284 (16)	0.0203 (12)	−0.0149 (15)
N1B	0.0194 (10)	0.0241 (11)	0.0129 (10)	−0.0010 (9)	−0.0013 (8)	0.0018 (9)
N2B	0.0213 (11)	0.0276 (12)	0.0166 (10)	−0.0040 (10)	−0.0060 (8)	0.0065 (10)
C1B	0.0229 (13)	0.0238 (13)	0.0121 (11)	−0.0016 (11)	−0.0008 (10)	0.0016 (10)
C2B	0.0225 (13)	0.0246 (14)	0.0192 (12)	−0.0029 (11)	−0.0022 (10)	0.0031 (11)
C3B	0.0272 (14)	0.0265 (14)	0.0201 (13)	−0.0085 (12)	−0.0031 (11)	0.0027 (12)
C4B	0.0303 (15)	0.0195 (13)	0.0168 (12)	−0.0020 (11)	0.0012 (10)	0.0028 (11)
C5B	0.0200 (12)	0.0309 (15)	0.0139 (12)	−0.0017 (11)	−0.0052 (10)	0.0085 (11)
C6B	0.0196 (13)	0.0294 (15)	0.0194 (13)	0.0022 (11)	−0.0052 (10)	0.0040 (12)
C7B	0.0237 (14)	0.0311 (15)	0.0232 (14)	0.0026 (12)	−0.0013 (11)	0.0059 (12)
C8B	0.0373 (17)	0.0284 (16)	0.0348 (17)	−0.0031 (14)	−0.0021 (13)	0.0093 (14)
C9B	0.0387 (18)	0.0281 (16)	0.0386 (18)	−0.0073 (13)	−0.0152 (14)	0.0030 (14)
C10B	0.055 (2)	0.042 (2)	0.0224 (15)	−0.0088 (17)	−0.0138 (14)	−0.0012 (15)
C11B	0.0391 (17)	0.0393 (18)	0.0221 (14)	−0.0101 (15)	−0.0080 (12)	0.0072 (14)
C12B	0.0222 (13)	0.0244 (14)	0.0268 (14)	−0.0006 (11)	0.0034 (11)	0.0080 (12)
C13B	0.0307 (15)	0.0326 (16)	0.0247 (15)	0.0033 (13)	0.0051 (12)	0.0032 (13)
C14B	0.0361 (17)	0.0317 (17)	0.0400 (17)	0.0029 (14)	0.0129 (14)	0.0000 (14)
C15B	0.0371 (18)	0.0255 (16)	0.064 (2)	0.0038 (13)	0.0119 (17)	0.0029 (16)
C16B	0.0293 (17)	0.0344 (18)	0.062 (2)	0.0083 (15)	−0.0050 (16)	0.0123 (18)
C17B	0.0352 (17)	0.0322 (16)	0.0320 (16)	−0.0005 (14)	−0.0057 (13)	0.0085 (14)
C18B	0.0203 (13)	0.0451 (19)	0.0305 (15)	−0.0038 (13)	−0.0087 (12)	0.0066 (14)
C19B	0.0205 (14)	0.047 (2)	0.051 (2)	0.0004 (14)	0.0002 (14)	0.0025 (17)
C20B	0.048 (2)	0.057 (3)	0.042 (2)	−0.0171 (18)	0.0182 (17)	−0.0066 (18)
C21B	0.050 (2)	0.043 (2)	0.0324 (17)	−0.0087 (17)	0.0099 (15)	−0.0010 (15)
O1C	0.0310 (10)	0.0296 (11)	0.0204 (10)	−0.0041 (9)	−0.0035 (8)	0.0087 (9)
O2C	0.0673 (18)	0.088 (2)	0.0270 (12)	−0.0519 (18)	−0.0058 (12)	0.0159 (14)
O3C	0.0692 (19)	0.0376 (15)	0.0648 (19)	−0.0111 (14)	−0.0002 (15)	−0.0087 (14)
O4C	0.0332 (11)	0.0427 (13)	0.0207 (10)	−0.0072 (10)	−0.0099 (8)	0.0117 (10)
O5C	0.0292 (11)	0.0374 (12)	0.0267 (10)	−0.0088 (9)	0.0004 (9)	0.0107 (10)
O6C	0.0218 (10)	0.0573 (16)	0.0278 (11)	−0.0154 (10)	−0.0049 (8)	0.0090 (11)
O7C	0.0406 (12)	0.0345 (12)	0.0165 (10)	−0.0071 (10)	−0.0020 (8)	0.0073 (9)
N1C	0.0459 (17)	0.0417 (17)	0.0293 (14)	−0.0153 (14)	−0.0128 (12)	0.0133 (13)
N2C	0.0233 (11)	0.0312 (13)	0.0216 (12)	−0.0020 (10)	0.0000 (9)	0.0068 (10)
N3C	0.0153 (10)	0.0310 (13)	0.0205 (11)	0.0015 (9)	0.0004 (9)	0.0040 (10)
C1C	0.0171 (12)	0.0275 (14)	0.0170 (12)	0.0016 (11)	0.0022 (10)	0.0032 (11)
C2C	0.0213 (13)	0.0305 (15)	0.0239 (14)	−0.0048 (12)	0.0002 (11)	0.0070 (12)
C3C	0.0211 (13)	0.0322 (16)	0.0200 (13)	−0.0013 (12)	−0.0050 (10)	0.0023 (12)
C4C	0.0201 (12)	0.0295 (15)	0.0173 (13)	−0.0002 (11)	0.0017 (10)	0.0060 (12)
C5C	0.0177 (12)	0.0261 (14)	0.0198 (13)	0.0001 (11)	0.0019 (10)	0.0052 (11)
C6C	0.0154 (12)	0.0303 (15)	0.0165 (12)	0.0031 (11)	0.0027 (10)	0.0029 (11)
O1D	0.0319 (11)	0.0273 (11)	0.0150 (9)	0.0069 (9)	−0.0009 (8)	0.0046 (8)
O2D	0.087 (2)	0.0286 (13)	0.0426 (14)	0.0205 (14)	−0.0201 (14)	0.0040 (12)
O3D	0.0560 (15)	0.0280 (12)	0.0408 (13)	−0.0048 (11)	−0.0066 (12)	−0.0063 (11)
O4D	0.0341 (11)	0.0426 (13)	0.0150 (9)	0.0040 (10)	−0.0027 (8)	0.0058 (9)
O5D	0.0449 (13)	0.0324 (12)	0.0346 (12)	−0.0054 (11)	−0.0050 (10)	0.0190 (10)
O6D	0.0595 (16)	0.0320 (13)	0.0415 (13)	−0.0186 (12)	0.0063 (12)	−0.0136 (11)
O7D	0.0487 (14)	0.0398 (13)	0.0205 (10)	−0.0008 (11)	0.0022 (9)	−0.0082 (10)
N1D	0.0437 (15)	0.0195 (13)	0.0218 (12)	0.0033 (11)	0.0013 (11)	0.0009 (10)
N2D	0.0164 (11)	0.0327 (13)	0.0197 (11)	0.0018 (9)	0.0003 (9)	0.0087 (10)
N3D	0.0227 (11)	0.0260 (13)	0.0225 (12)	0.0014 (10)	−0.0033 (9)	−0.0054 (10)
C1D	0.0131 (11)	0.0218 (13)	0.0127 (11)	0.0025 (10)	−0.0003 (9)	0.0024 (10)
C2D	0.0204 (12)	0.0190 (13)	0.0197 (13)	0.0033 (10)	0.0000 (10)	0.0009 (11)
C3D	0.0147 (11)	0.0250 (13)	0.0136 (12)	0.0034 (10)	−0.0006 (9)	−0.0012 (10)
C4D	0.0117 (11)	0.0259 (14)	0.0162 (12)	0.0020 (10)	−0.0002 (9)	0.0067 (11)
C5D	0.0124 (11)	0.0199 (13)	0.0231 (13)	0.0015 (10)	0.0003 (9)	0.0059 (11)
C6D	0.0125 (11)	0.0229 (13)	0.0194 (13)	0.0016 (10)	−0.0007 (9)	−0.0043 (11)
O1E	0.0384 (11)	0.0251 (11)	0.0117 (9)	0.0039 (9)	0.0000 (8)	0.0020 (8)
O2E	0.0682 (18)	0.0368 (14)	0.0428 (14)	0.0218 (13)	−0.0140 (13)	−0.0115 (12)
O3E	0.0667 (18)	0.0423 (15)	0.0358 (13)	−0.0032 (13)	−0.0139 (12)	−0.0175 (12)
O4E	0.0369 (13)	0.081 (2)	0.0165 (11)	0.0089 (13)	−0.0002 (9)	0.0123 (12)
O5E	0.0317 (12)	0.0609 (18)	0.0396 (13)	−0.0068 (12)	−0.0052 (10)	0.0312 (13)
O6E	0.112 (3)	0.0382 (16)	0.0483 (16)	−0.0372 (18)	0.0095 (17)	−0.0060 (13)
O7E	0.0512 (14)	0.0322 (12)	0.0151 (9)	−0.0055 (10)	−0.0006 (9)	−0.0010 (9)
N1E	0.0437 (16)	0.0342 (15)	0.0232 (13)	0.0069 (12)	−0.0033 (11)	−0.0115 (12)
N2E	0.0125 (11)	0.065 (2)	0.0231 (13)	0.0012 (12)	0.0016 (9)	0.0171 (14)
N3E	0.0319 (13)	0.0232 (13)	0.0275 (13)	−0.0049 (11)	−0.0001 (10)	−0.0021 (11)
C1E	0.0149 (12)	0.0272 (14)	0.0158 (12)	0.0035 (10)	0.0002 (9)	0.0007 (11)
C2E	0.0188 (12)	0.0268 (15)	0.0196 (13)	0.0040 (10)	0.0014 (10)	−0.0028 (11)
C3E	0.0139 (12)	0.0486 (19)	0.0121 (12)	0.0033 (12)	0.0019 (9)	−0.0049 (12)
C4E	0.0131 (12)	0.0466 (19)	0.0184 (13)	0.0025 (12)	0.0025 (10)	0.0110 (13)
C5E	0.0154 (12)	0.0302 (15)	0.0258 (14)	−0.0018 (11)	0.0009 (10)	0.0093 (12)
C6E	0.0154 (12)	0.0252 (14)	0.0177 (12)	−0.0016 (10)	0.0001 (10)	0.0013 (11)
O1F	0.0262 (10)	0.0294 (11)	0.0217 (10)	0.0024 (8)	−0.0022 (8)	0.0094 (9)
O2F	0.0445 (14)	0.0596 (18)	0.0400 (14)	−0.0253 (13)	0.0084 (11)	0.0029 (13)
O3F	0.086 (2)	0.067 (2)	0.064 (2)	−0.0362 (19)	0.0176 (17)	−0.0337 (18)
O4F	0.0310 (11)	0.0461 (13)	0.0183 (10)	−0.0084 (10)	−0.0061 (8)	0.0079 (9)
O5F	0.0220 (10)	0.0427 (13)	0.0267 (10)	−0.0079 (9)	0.0027 (8)	0.0118 (10)
O6F	0.0215 (11)	0.0641 (17)	0.0295 (11)	−0.0136 (11)	−0.0049 (9)	0.0105 (12)
O7F	0.0427 (13)	0.0410 (13)	0.0178 (10)	−0.0085 (11)	−0.0045 (9)	0.0087 (10)
N1F	0.0380 (14)	0.0319 (14)	0.0192 (12)	−0.0092 (12)	−0.0045 (10)	0.0053 (11)
N2F	0.0176 (11)	0.0341 (13)	0.0176 (11)	−0.0002 (10)	0.0032 (9)	0.0041 (10)
N3F	0.0193 (11)	0.0366 (14)	0.0188 (11)	0.0008 (10)	−0.0008 (9)	0.0031 (11)
C1F	0.0178 (12)	0.0282 (14)	0.0150 (12)	0.0058 (11)	0.0053 (10)	0.0028 (11)
C2F	0.0218 (13)	0.0252 (14)	0.0189 (12)	−0.0002 (11)	0.0029 (10)	−0.0022 (11)
C3F	0.0194 (12)	0.0297 (14)	0.0137 (11)	0.0020 (11)	0.0011 (9)	0.0000 (11)
C4F	0.0142 (11)	0.0311 (15)	0.0162 (12)	0.0044 (11)	0.0037 (9)	0.0035 (11)
C5F	0.0143 (11)	0.0328 (15)	0.0189 (13)	0.0021 (11)	0.0034 (10)	0.0019 (12)
C6F	0.0145 (12)	0.0311 (15)	0.0202 (13)	0.0043 (11)	0.0003 (10)	0.0020 (12)

### Geometric parameters (Å, °)

**Table:**

Cl1A—C9A	1.745 (3)	C11B—H11B	0.9500
O1A—C20A	1.413 (4)	C12B—C17B	1.389 (4)
O1A—C19A	1.425 (4)	C12B—C13B	1.400 (4)
O2A—C21A	1.306 (4)	C13B—C14B	1.394 (5)
O2A—H2AD	0.8400	C13B—H13B	0.9500
O3A—C21A	1.216 (4)	C14B—C15B	1.382 (5)
N1A—C4A	1.502 (3)	C14B—H14B	0.9500
N1A—C1A	1.512 (3)	C15B—C16B	1.390 (6)
N1A—C5A	1.526 (3)	C15B—H15B	0.9500
N1A—H1AC	0.9300	C16B—C17B	1.376 (5)
N2A—C3A	1.495 (4)	C16B—H16B	0.9500
N2A—C2A	1.495 (3)	C17B—H17B	0.9500
N2A—C18A	1.510 (3)	C18B—C19B	1.510 (5)
N2A—H2AC	0.9300	C18B—H18C	0.9900
C1A—C2A	1.513 (3)	C18B—H18D	0.9900
C1A—H1AA	0.9900	C19B—H19C	0.9900
C1A—H1AB	0.9900	C19B—H19D	0.9900
C2A—H2AA	0.9900	C20B—C21B	1.505 (5)
C2A—H2AB	0.9900	C20B—H20C	0.9900
C3A—C4A	1.512 (3)	C20B—H20D	0.9900
C3A—H3AA	0.9900	O1C—C1C	1.248 (4)
C3A—H3AB	0.9900	O2C—N1C	1.213 (4)
C4A—H4AA	0.9900	O3C—N1C	1.224 (4)
C4A—H4AB	0.9900	O4C—N2C	1.228 (3)
C5A—C6A	1.514 (4)	O5C—N2C	1.239 (3)
C5A—C12A	1.525 (4)	O6C—N3C	1.236 (3)
C5A—H5AA	1.0000	O7C—N3C	1.220 (3)
C6A—C7A	1.398 (4)	N1C—C2C	1.467 (4)
C6A—C11A	1.399 (4)	N2C—C4C	1.449 (4)
C7A—C8A	1.386 (4)	N3C—C6C	1.448 (4)
C7A—H7AA	0.9500	C1C—C6C	1.444 (4)
C8A—C9A	1.381 (5)	C1C—C2C	1.448 (4)
C8A—H8AA	0.9500	C2C—C3C	1.360 (4)
C9A—C10A	1.376 (4)	C3C—C4C	1.398 (4)
C10A—C11A	1.393 (4)	C3C—H3CA	0.9500
C10A—H10A	0.9500	C4C—C5C	1.378 (4)
C11A—H11A	0.9500	C5C—C6C	1.386 (4)
C12A—C17A	1.390 (4)	C5C—H5CA	0.9500
C12A—C13A	1.400 (4)	O1D—C1D	1.246 (3)
C13A—C14A	1.390 (5)	O2D—N1D	1.238 (4)
C13A—H13A	0.9500	O3D—N1D	1.218 (4)
C14A—C15A	1.390 (5)	O4D—N2D	1.232 (3)
C14A—H14A	0.9500	O5D—N2D	1.235 (4)
C15A—C16A	1.394 (5)	O6D—N3D	1.228 (4)
C15A—H15A	0.9500	O7D—N3D	1.222 (3)
C16A—C17A	1.387 (5)	N1D—C2D	1.460 (4)
C16A—H16A	0.9500	N2D—C4D	1.443 (3)
C17A—H17A	0.9500	N3D—C6D	1.460 (4)
C18A—C19A	1.494 (4)	C1D—C6D	1.442 (4)
C18A—H18A	0.9900	C1D—C2D	1.454 (4)
C18A—H18B	0.9900	C2D—C3D	1.368 (4)
C19A—H19A	0.9900	C3D—C4D	1.384 (4)
C19A—H19B	0.9900	C3D—H3DA	0.9500
C20A—C21A	1.493 (5)	C4D—C5D	1.388 (4)
C20A—H20A	0.9900	C5D—C6D	1.368 (4)
C20A—H20B	0.9900	C5D—H5DA	0.9500
Cl1B—C9B	1.739 (3)	O1E—C1E	1.249 (3)
O1B—C20B	1.410 (4)	O2E—N1E	1.227 (4)
O1B—C19B	1.419 (4)	O3E—N1E	1.232 (4)
O2B—C21B	1.211 (4)	O4E—N2E	1.235 (4)
O3B—C21B	1.304 (4)	O5E—N2E	1.235 (4)
O3B—H3BC	0.8400	O6E—N3E	1.227 (4)
N1B—C4B	1.505 (4)	O7E—N3E	1.212 (3)
N1B—C1B	1.512 (4)	N1E—C2E	1.451 (4)
N1B—C5B	1.535 (3)	N2E—C4E	1.441 (4)
N1B—H1BC	0.9300	N3E—C6E	1.454 (4)
N2B—C3B	1.481 (4)	C1E—C6E	1.442 (4)
N2B—C2B	1.499 (4)	C1E—C2E	1.454 (4)
N2B—C18B	1.505 (4)	C2E—C3E	1.368 (4)
N2B—H2BC	0.9300	C3E—C4E	1.382 (5)
C1B—C2B	1.512 (4)	C3E—H3EA	0.9500
C1B—H1BA	0.9900	C4E—C5E	1.387 (5)
C1B—H1BB	0.9900	C5E—C6E	1.383 (4)
C2B—H2BA	0.9900	C5E—H5EA	0.9500
C2B—H2BB	0.9900	O1F—C1F	1.231 (4)
C3B—C4B	1.508 (4)	O2F—N1F	1.206 (4)
C3B—H3BA	0.9900	O3F—N1F	1.205 (4)
C3B—H3BB	0.9900	O4F—N2F	1.228 (3)
C4B—H4BA	0.9900	O5F—N2F	1.233 (3)
C4B—H4BB	0.9900	O6F—N3F	1.224 (4)
C5B—C12B	1.514 (4)	O7F—N3F	1.228 (3)
C5B—C6B	1.520 (4)	N1F—C2F	1.465 (4)
C5B—H5BA	1.0000	N2F—C4F	1.443 (4)
C6B—C7B	1.386 (4)	N3F—C6F	1.462 (4)
C6B—C11B	1.390 (4)	C1F—C2F	1.453 (4)
C7B—C8B	1.388 (5)	C1F—C6F	1.453 (4)
C7B—H7BA	0.9500	C2F—C3F	1.361 (4)
C8B—C9B	1.394 (5)	C3F—C4F	1.399 (4)
C8B—H8BA	0.9500	C3F—H3FA	0.9500
C9B—C10B	1.367 (5)	C4F—C5F	1.386 (4)
C10B—C11B	1.388 (5)	C5F—C6F	1.372 (4)
C10B—H10B	0.9500	C5F—H5FA	0.9500
			
C20A—O1A—C19A	114.4 (3)	C10B—C9B—Cl1B	117.9 (3)
C21A—O2A—H2AD	109.5	C8B—C9B—Cl1B	120.2 (3)
C4A—N1A—C1A	110.86 (19)	C9B—C10B—C11B	118.8 (3)
C4A—N1A—C5A	108.93 (19)	C9B—C10B—H10B	120.6
C1A—N1A—C5A	109.52 (19)	C11B—C10B—H10B	120.6
C4A—N1A—H1AC	109.2	C10B—C11B—C6B	120.6 (3)
C1A—N1A—H1AC	109.2	C10B—C11B—H11B	119.7
C5A—N1A—H1AC	109.2	C6B—C11B—H11B	119.7
C3A—N2A—C2A	106.79 (19)	C17B—C12B—C13B	118.5 (3)
C3A—N2A—C18A	113.3 (2)	C17B—C12B—C5B	116.1 (3)
C2A—N2A—C18A	111.2 (2)	C13B—C12B—C5B	125.3 (3)
C3A—N2A—H2AC	108.5	C14B—C13B—C12B	120.0 (3)
C2A—N2A—H2AC	108.5	C14B—C13B—H13B	120.0
C18A—N2A—H2AC	108.5	C12B—C13B—H13B	120.0
N1A—C1A—C2A	111.4 (2)	C15B—C14B—C13B	120.7 (3)
N1A—C1A—H1AA	109.3	C15B—C14B—H14B	119.7
C2A—C1A—H1AA	109.3	C13B—C14B—H14B	119.7
N1A—C1A—H1AB	109.3	C14B—C15B—C16B	119.2 (3)
C2A—C1A—H1AB	109.3	C14B—C15B—H15B	120.4
H1AA—C1A—H1AB	108.0	C16B—C15B—H15B	120.4
N2A—C2A—C1A	110.2 (2)	C17B—C16B—C15B	120.5 (3)
N2A—C2A—H2AA	109.6	C17B—C16B—H16B	119.8
C1A—C2A—H2AA	109.6	C15B—C16B—H16B	119.8
N2A—C2A—H2AB	109.6	C16B—C17B—C12B	121.2 (3)
C1A—C2A—H2AB	109.6	C16B—C17B—H17B	119.4
H2AA—C2A—H2AB	108.1	C12B—C17B—H17B	119.4
N2A—C3A—C4A	110.6 (2)	N2B—C18B—C19B	111.7 (3)
N2A—C3A—H3AA	109.5	N2B—C18B—H18C	109.3
C4A—C3A—H3AA	109.5	C19B—C18B—H18C	109.3
N2A—C3A—H3AB	109.5	N2B—C18B—H18D	109.3
C4A—C3A—H3AB	109.5	C19B—C18B—H18D	109.3
H3AA—C3A—H3AB	108.1	H18C—C18B—H18D	107.9
N1A—C4A—C3A	112.1 (2)	O1B—C19B—C18B	106.6 (3)
N1A—C4A—H4AA	109.2	O1B—C19B—H19C	110.4
C3A—C4A—H4AA	109.2	C18B—C19B—H19C	110.4
N1A—C4A—H4AB	109.2	O1B—C19B—H19D	110.4
C3A—C4A—H4AB	109.2	C18B—C19B—H19D	110.4
H4AA—C4A—H4AB	107.9	H19C—C19B—H19D	108.6
C6A—C5A—C12A	111.0 (2)	O1B—C20B—C21B	111.6 (3)
C6A—C5A—N1A	114.1 (2)	O1B—C20B—H20C	109.3
C12A—C5A—N1A	109.8 (2)	C21B—C20B—H20C	109.3
C6A—C5A—H5AA	107.2	O1B—C20B—H20D	109.3
C12A—C5A—H5AA	107.2	C21B—C20B—H20D	109.3
N1A—C5A—H5AA	107.2	H20C—C20B—H20D	108.0
C7A—C6A—C11A	119.3 (3)	O2B—C21B—O3B	124.9 (4)
C7A—C6A—C5A	116.2 (2)	O2B—C21B—C20B	122.3 (3)
C11A—C6A—C5A	124.4 (2)	O3B—C21B—C20B	112.8 (3)
C8A—C7A—C6A	120.5 (3)	O2C—N1C—O3C	124.7 (3)
C8A—C7A—H7AA	119.8	O2C—N1C—C2C	118.0 (3)
C6A—C7A—H7AA	119.8	O3C—N1C—C2C	117.3 (3)
C9A—C8A—C7A	118.9 (3)	O4C—N2C—O5C	123.3 (2)
C9A—C8A—H8AA	120.6	O4C—N2C—C4C	118.6 (2)
C7A—C8A—H8AA	120.6	O5C—N2C—C4C	118.1 (2)
C10A—C9A—C8A	122.1 (3)	O7C—N3C—O6C	122.2 (2)
C10A—C9A—Cl1A	119.2 (2)	O7C—N3C—C6C	119.6 (2)
C8A—C9A—Cl1A	118.7 (2)	O6C—N3C—C6C	118.2 (2)
C9A—C10A—C11A	118.9 (3)	O1C—C1C—C6C	126.6 (2)
C9A—C10A—H10A	120.5	O1C—C1C—C2C	121.6 (3)
C11A—C10A—H10A	120.5	C6C—C1C—C2C	111.6 (2)
C10A—C11A—C6A	120.2 (3)	C3C—C2C—C1C	126.1 (3)
C10A—C11A—H11A	119.9	C3C—C2C—N1C	118.0 (3)
C6A—C11A—H11A	119.9	C1C—C2C—N1C	115.8 (3)
C17A—C12A—C13A	119.5 (3)	C2C—C3C—C4C	117.7 (3)
C17A—C12A—C5A	120.8 (2)	C2C—C3C—H3CA	121.2
C13A—C12A—C5A	119.6 (2)	C4C—C3C—H3CA	121.2
C14A—C13A—C12A	120.1 (3)	C5C—C4C—C3C	121.5 (3)
C14A—C13A—H13A	119.9	C5C—C4C—N2C	119.7 (3)
C12A—C13A—H13A	119.9	C3C—C4C—N2C	118.8 (2)
C13A—C14A—C15A	120.4 (3)	C4C—C5C—C6C	119.6 (3)
C13A—C14A—H14A	119.8	C4C—C5C—H5CA	120.2
C15A—C14A—H14A	119.8	C6C—C5C—H5CA	120.2
C14A—C15A—C16A	119.1 (3)	C5C—C6C—C1C	123.5 (2)
C14A—C15A—H15A	120.4	C5C—C6C—N3C	116.9 (3)
C16A—C15A—H15A	120.4	C1C—C6C—N3C	119.6 (2)
C17A—C16A—C15A	120.9 (3)	O3D—N1D—O2D	123.9 (3)
C17A—C16A—H16A	119.5	O3D—N1D—C2D	118.2 (3)
C15A—C16A—H16A	119.5	O2D—N1D—C2D	117.6 (3)
C16A—C17A—C12A	119.9 (3)	O4D—N2D—O5D	122.6 (2)
C16A—C17A—H17A	120.1	O4D—N2D—C4D	119.2 (3)
C12A—C17A—H17A	120.1	O5D—N2D—C4D	118.2 (3)
C19A—C18A—N2A	111.9 (2)	O7D—N3D—O6D	124.0 (3)
C19A—C18A—H18A	109.2	O7D—N3D—C6D	119.3 (3)
N2A—C18A—H18A	109.2	O6D—N3D—C6D	116.7 (2)
C19A—C18A—H18B	109.2	O1D—C1D—C6D	126.1 (2)
N2A—C18A—H18B	109.2	O1D—C1D—C2D	122.3 (3)
H18A—C18A—H18B	107.9	C6D—C1D—C2D	111.6 (2)
O1A—C19A—C18A	108.0 (3)	C3D—C2D—C1D	124.7 (3)
O1A—C19A—H19A	110.1	C3D—C2D—N1D	116.3 (3)
C18A—C19A—H19A	110.1	C1D—C2D—N1D	119.1 (2)
O1A—C19A—H19B	110.1	C2D—C3D—C4D	118.8 (3)
C18A—C19A—H19B	110.1	C2D—C3D—H3DA	120.6
H19A—C19A—H19B	108.4	C4D—C3D—H3DA	120.6
O1A—C20A—C21A	111.4 (3)	C3D—C4D—C5D	121.2 (2)
O1A—C20A—H20A	109.3	C3D—C4D—N2D	118.5 (3)
C21A—C20A—H20A	109.3	C5D—C4D—N2D	120.3 (3)
O1A—C20A—H20B	109.3	C6D—C5D—C4D	119.2 (3)
C21A—C20A—H20B	109.3	C6D—C5D—H5DA	120.4
H20A—C20A—H20B	108.0	C4D—C5D—H5DA	120.4
O3A—C21A—O2A	123.8 (3)	C5D—C6D—C1D	124.5 (3)
O3A—C21A—C20A	122.7 (3)	C5D—C6D—N3D	116.6 (3)
O2A—C21A—C20A	113.4 (3)	C1D—C6D—N3D	118.9 (2)
C20B—O1B—C19B	113.8 (3)	O2E—N1E—O3E	123.4 (3)
C21B—O3B—H3BC	109.5	O2E—N1E—C2E	118.7 (3)
C4B—N1B—C1B	110.2 (2)	O3E—N1E—C2E	117.8 (3)
C4B—N1B—C5B	110.7 (2)	O5E—N2E—O4E	123.1 (3)
C1B—N1B—C5B	108.4 (2)	O5E—N2E—C4E	119.6 (3)
C4B—N1B—H1BC	109.2	O4E—N2E—C4E	117.4 (3)
C1B—N1B—H1BC	109.2	O7E—N3E—O6E	122.7 (3)
C5B—N1B—H1BC	109.2	O7E—N3E—C6E	120.5 (2)
C3B—N2B—C2B	106.7 (2)	O6E—N3E—C6E	116.8 (3)
C3B—N2B—C18B	111.2 (2)	O1E—C1E—C6E	125.9 (3)
C2B—N2B—C18B	113.0 (2)	O1E—C1E—C2E	122.5 (3)
C3B—N2B—H2BC	108.6	C6E—C1E—C2E	111.6 (2)
C2B—N2B—H2BC	108.6	C3E—C2E—N1E	116.4 (3)
C18B—N2B—H2BC	108.6	C3E—C2E—C1E	124.5 (3)
N1B—C1B—C2B	112.9 (2)	N1E—C2E—C1E	119.1 (3)
N1B—C1B—H1BA	109.0	C2E—C3E—C4E	119.1 (3)
C2B—C1B—H1BA	109.0	C2E—C3E—H3EA	120.4
N1B—C1B—H1BB	109.0	C4E—C3E—H3EA	120.4
C2B—C1B—H1BB	109.0	C3E—C4E—C5E	121.4 (3)
H1BA—C1B—H1BB	107.8	C3E—C4E—N2E	119.4 (3)
N2B—C2B—C1B	109.9 (2)	C5E—C4E—N2E	119.2 (3)
N2B—C2B—H2BA	109.7	C6E—C5E—C4E	118.6 (3)
C1B—C2B—H2BA	109.7	C6E—C5E—H5EA	120.7
N2B—C2B—H2BB	109.7	C4E—C5E—H5EA	120.7
C1B—C2B—H2BB	109.7	C5E—C6E—C1E	124.5 (3)
H2BA—C2B—H2BB	108.2	C5E—C6E—N3E	116.3 (3)
N2B—C3B—C4B	111.3 (2)	C1E—C6E—N3E	119.1 (2)
N2B—C3B—H3BA	109.4	O3F—N1F—O2F	124.5 (3)
C4B—C3B—H3BA	109.4	O3F—N1F—C2F	117.5 (3)
N2B—C3B—H3BB	109.4	O2F—N1F—C2F	117.9 (3)
C4B—C3B—H3BB	109.4	O4F—N2F—O5F	123.4 (2)
H3BA—C3B—H3BB	108.0	O4F—N2F—C4F	118.2 (2)
N1B—C4B—C3B	112.1 (2)	O5F—N2F—C4F	118.5 (2)
N1B—C4B—H4BA	109.2	O6F—N3F—O7F	122.5 (2)
C3B—C4B—H4BA	109.2	O6F—N3F—C6F	118.9 (2)
N1B—C4B—H4BB	109.2	O7F—N3F—C6F	118.6 (2)
C3B—C4B—H4BB	109.2	O1F—C1F—C2F	121.4 (3)
H4BA—C4B—H4BB	107.9	O1F—C1F—C6F	127.6 (3)
C12B—C5B—C6B	112.3 (2)	C2F—C1F—C6F	110.9 (2)
C12B—C5B—N1B	114.7 (2)	C3F—C2F—C1F	126.2 (3)
C6B—C5B—N1B	109.2 (2)	C3F—C2F—N1F	118.0 (2)
C12B—C5B—H5BA	106.7	C1F—C2F—N1F	115.8 (2)
C6B—C5B—H5BA	106.7	C2F—C3F—C4F	118.0 (2)
N1B—C5B—H5BA	106.7	C2F—C3F—H3FA	121.0
C7B—C6B—C11B	119.8 (3)	C4F—C3F—H3FA	121.0
C7B—C6B—C5B	120.8 (3)	C5F—C4F—C3F	120.7 (3)
C11B—C6B—C5B	119.4 (3)	C5F—C4F—N2F	120.3 (3)
C6B—C7B—C8B	120.0 (3)	C3F—C4F—N2F	119.0 (2)
C6B—C7B—H7BA	120.0	C6F—C5F—C4F	120.1 (3)
C8B—C7B—H7BA	120.0	C6F—C5F—H5FA	119.9
C7B—C8B—C9B	118.9 (3)	C4F—C5F—H5FA	119.9
C7B—C8B—H8BA	120.5	C5F—C6F—C1F	123.9 (3)
C9B—C8B—H8BA	120.5	C5F—C6F—N3F	116.4 (3)
C10B—C9B—C8B	121.8 (3)	C1F—C6F—N3F	119.7 (2)
			
C4A—N1A—C1A—C2A	51.3 (3)	N1C—C2C—C3C—C4C	176.6 (3)
C5A—N1A—C1A—C2A	171.5 (2)	C2C—C3C—C4C—C5C	−1.0 (4)
C3A—N2A—C2A—C1A	63.6 (3)	C2C—C3C—C4C—N2C	−178.7 (3)
C18A—N2A—C2A—C1A	−172.3 (2)	O4C—N2C—C4C—C5C	173.5 (3)
N1A—C1A—C2A—N2A	−59.0 (3)	O5C—N2C—C4C—C5C	−6.7 (4)
C2A—N2A—C3A—C4A	−62.8 (3)	O4C—N2C—C4C—C3C	−8.7 (4)
C18A—N2A—C3A—C4A	174.4 (2)	O5C—N2C—C4C—C3C	171.1 (3)
C1A—N1A—C4A—C3A	−50.6 (3)	C3C—C4C—C5C—C6C	2.3 (4)
C5A—N1A—C4A—C3A	−171.2 (2)	N2C—C4C—C5C—C6C	180.0 (3)
N2A—C3A—C4A—N1A	57.6 (3)	C4C—C5C—C6C—C1C	−2.8 (4)
C4A—N1A—C5A—C6A	177.9 (2)	C4C—C5C—C6C—N3C	178.3 (3)
C1A—N1A—C5A—C6A	56.5 (3)	O1C—C1C—C6C—C5C	−174.3 (3)
C4A—N1A—C5A—C12A	−56.7 (3)	C2C—C1C—C6C—C5C	2.0 (4)
C1A—N1A—C5A—C12A	−178.1 (2)	O1C—C1C—C6C—N3C	4.5 (4)
C12A—C5A—C6A—C7A	82.4 (3)	C2C—C1C—C6C—N3C	−179.2 (2)
N1A—C5A—C6A—C7A	−152.8 (2)	O7C—N3C—C6C—C5C	−164.6 (3)
C12A—C5A—C6A—C11A	−93.1 (3)	O6C—N3C—C6C—C5C	14.7 (4)
N1A—C5A—C6A—C11A	31.6 (4)	O7C—N3C—C6C—C1C	16.5 (4)
C11A—C6A—C7A—C8A	2.4 (4)	O6C—N3C—C6C—C1C	−164.2 (3)
C5A—C6A—C7A—C8A	−173.4 (3)	O1D—C1D—C2D—C3D	176.3 (3)
C6A—C7A—C8A—C9A	−1.0 (4)	C6D—C1D—C2D—C3D	−3.6 (4)
C7A—C8A—C9A—C10A	−1.8 (4)	O1D—C1D—C2D—N1D	−3.5 (4)
C7A—C8A—C9A—Cl1A	176.2 (2)	C6D—C1D—C2D—N1D	176.7 (3)
C8A—C9A—C10A—C11A	3.2 (5)	O3D—N1D—C2D—C3D	−34.6 (4)
Cl1A—C9A—C10A—C11A	−174.8 (2)	O2D—N1D—C2D—C3D	140.2 (3)
C9A—C10A—C11A—C6A	−1.7 (4)	O3D—N1D—C2D—C1D	145.1 (3)
C7A—C6A—C11A—C10A	−1.0 (4)	O2D—N1D—C2D—C1D	−40.0 (4)
C5A—C6A—C11A—C10A	174.4 (3)	C1D—C2D—C3D—C4D	4.6 (4)
C6A—C5A—C12A—C17A	54.8 (3)	N1D—C2D—C3D—C4D	−175.7 (3)
N1A—C5A—C12A—C17A	−72.3 (3)	C2D—C3D—C4D—C5D	−1.7 (4)
C6A—C5A—C12A—C13A	−123.5 (3)	C2D—C3D—C4D—N2D	179.5 (2)
N1A—C5A—C12A—C13A	109.4 (3)	O4D—N2D—C4D—C3D	3.0 (4)
C17A—C12A—C13A—C14A	0.3 (5)	O5D—N2D—C4D—C3D	−177.0 (3)
C5A—C12A—C13A—C14A	178.6 (3)	O4D—N2D—C4D—C5D	−175.8 (2)
C12A—C13A—C14A—C15A	−1.1 (5)	O5D—N2D—C4D—C5D	4.3 (4)
C13A—C14A—C15A—C16A	1.3 (6)	C3D—C4D—C5D—C6D	−1.7 (4)
C14A—C15A—C16A—C17A	−0.5 (5)	N2D—C4D—C5D—C6D	177.0 (2)
C15A—C16A—C17A—C12A	−0.3 (5)	C4D—C5D—C6D—C1D	2.6 (4)
C13A—C12A—C17A—C16A	0.5 (4)	C4D—C5D—C6D—N3D	−179.1 (2)
C5A—C12A—C17A—C16A	−177.8 (2)	O1D—C1D—C6D—C5D	−179.9 (2)
C3A—N2A—C18A—C19A	−72.7 (3)	C2D—C1D—C6D—C5D	−0.1 (4)
C2A—N2A—C18A—C19A	167.0 (2)	O1D—C1D—C6D—N3D	1.8 (4)
C20A—O1A—C19A—C18A	159.0 (3)	C2D—C1D—C6D—N3D	−178.4 (2)
N2A—C18A—C19A—O1A	−60.1 (3)	O7D—N3D—C6D—C5D	154.8 (3)
C19A—O1A—C20A—C21A	−82.5 (4)	O6D—N3D—C6D—C5D	−23.9 (4)
O1A—C20A—C21A—O3A	19.3 (5)	O7D—N3D—C6D—C1D	−26.8 (4)
O1A—C20A—C21A—O2A	−161.1 (3)	O6D—N3D—C6D—C1D	154.5 (3)
C4B—N1B—C1B—C2B	50.0 (3)	O2E—N1E—C2E—C3E	−140.4 (3)
C5B—N1B—C1B—C2B	171.3 (2)	O3E—N1E—C2E—C3E	37.7 (4)
C3B—N2B—C2B—C1B	62.5 (3)	O2E—N1E—C2E—C1E	39.0 (4)
C18B—N2B—C2B—C1B	−174.9 (2)	O3E—N1E—C2E—C1E	−143.0 (3)
N1B—C1B—C2B—N2B	−57.8 (3)	O1E—C1E—C2E—C3E	−175.0 (3)
C2B—N2B—C3B—C4B	−63.3 (3)	C6E—C1E—C2E—C3E	3.3 (4)
C18B—N2B—C3B—C4B	173.0 (2)	O1E—C1E—C2E—N1E	5.7 (4)
C1B—N1B—C4B—C3B	−49.4 (3)	C6E—C1E—C2E—N1E	−175.9 (3)
C5B—N1B—C4B—C3B	−169.4 (2)	N1E—C2E—C3E—C4E	176.0 (3)
N2B—C3B—C4B—N1B	58.2 (3)	C1E—C2E—C3E—C4E	−3.3 (4)
C4B—N1B—C5B—C12B	−58.4 (3)	C2E—C3E—C4E—C5E	0.8 (4)
C1B—N1B—C5B—C12B	−179.4 (2)	C2E—C3E—C4E—N2E	179.8 (2)
C4B—N1B—C5B—C6B	174.6 (2)	O5E—N2E—C4E—C3E	−176.0 (3)
C1B—N1B—C5B—C6B	53.6 (3)	O4E—N2E—C4E—C3E	2.6 (4)
C12B—C5B—C6B—C7B	−58.2 (3)	O5E—N2E—C4E—C5E	3.0 (4)
N1B—C5B—C6B—C7B	70.2 (3)	O4E—N2E—C4E—C5E	−178.3 (3)
C12B—C5B—C6B—C11B	123.4 (3)	C3E—C4E—C5E—C6E	1.3 (4)
N1B—C5B—C6B—C11B	−108.2 (3)	N2E—C4E—C5E—C6E	−177.7 (2)
C11B—C6B—C7B—C8B	3.0 (5)	C4E—C5E—C6E—C1E	−1.1 (4)
C5B—C6B—C7B—C8B	−175.3 (3)	C4E—C5E—C6E—N3E	−179.7 (2)
C6B—C7B—C8B—C9B	−0.7 (5)	O1E—C1E—C6E—C5E	177.2 (3)
C7B—C8B—C9B—C10B	−1.9 (5)	C2E—C1E—C6E—C5E	−1.1 (4)
C7B—C8B—C9B—Cl1B	176.1 (3)	O1E—C1E—C6E—N3E	−4.2 (4)
C8B—C9B—C10B—C11B	2.2 (6)	C2E—C1E—C6E—N3E	177.5 (2)
Cl1B—C9B—C10B—C11B	−175.9 (3)	O7E—N3E—C6E—C5E	−157.0 (3)
C9B—C10B—C11B—C6B	0.2 (6)	O6E—N3E—C6E—C5E	23.5 (4)
C7B—C6B—C11B—C10B	−2.8 (5)	O7E—N3E—C6E—C1E	24.3 (4)
C5B—C6B—C11B—C10B	175.6 (3)	O6E—N3E—C6E—C1E	−155.2 (3)
C6B—C5B—C12B—C17B	−75.6 (3)	O1F—C1F—C2F—C3F	−173.2 (3)
N1B—C5B—C12B—C17B	159.0 (3)	C6F—C1F—C2F—C3F	5.0 (4)
C6B—C5B—C12B—C13B	103.1 (3)	O1F—C1F—C2F—N1F	5.8 (4)
N1B—C5B—C12B—C13B	−22.3 (4)	C6F—C1F—C2F—N1F	−175.9 (2)
C17B—C12B—C13B—C14B	−0.8 (5)	O3F—N1F—C2F—C3F	57.0 (4)
C5B—C12B—C13B—C14B	−179.5 (3)	O2F—N1F—C2F—C3F	−123.1 (3)
C12B—C13B—C14B—C15B	2.8 (5)	O3F—N1F—C2F—C1F	−122.1 (3)
C13B—C14B—C15B—C16B	−2.6 (5)	O2F—N1F—C2F—C1F	57.7 (4)
C14B—C15B—C16B—C17B	0.4 (6)	C1F—C2F—C3F—C4F	−3.7 (4)
C15B—C16B—C17B—C12B	1.6 (6)	N1F—C2F—C3F—C4F	177.3 (3)
C13B—C12B—C17B—C16B	−1.4 (5)	C2F—C3F—C4F—C5F	0.8 (4)
C5B—C12B—C17B—C16B	177.4 (3)	C2F—C3F—C4F—N2F	−178.2 (2)
C3B—N2B—C18B—C19B	−166.4 (3)	O4F—N2F—C4F—C5F	−175.7 (3)
C2B—N2B—C18B—C19B	73.6 (3)	O5F—N2F—C4F—C5F	4.9 (4)
C20B—O1B—C19B—C18B	−158.3 (3)	O4F—N2F—C4F—C3F	3.4 (4)
N2B—C18B—C19B—O1B	61.1 (4)	O5F—N2F—C4F—C3F	−176.1 (3)
C19B—O1B—C20B—C21B	84.1 (4)	C3F—C4F—C5F—C6F	0.1 (4)
O1B—C20B—C21B—O2B	−14.5 (6)	N2F—C4F—C5F—C6F	179.1 (2)
O1B—C20B—C21B—O3B	165.5 (4)	C4F—C5F—C6F—C1F	1.8 (4)
O1C—C1C—C2C—C3C	175.9 (3)	C4F—C5F—C6F—N3F	−177.8 (3)
C6C—C1C—C2C—C3C	−0.7 (4)	O1F—C1F—C6F—C5F	174.2 (3)
O1C—C1C—C2C—N1C	−0.6 (4)	C2F—C1F—C6F—C5F	−4.0 (4)
C6C—C1C—C2C—N1C	−177.2 (3)	O1F—C1F—C6F—N3F	−6.2 (4)
O2C—N1C—C2C—C3C	127.6 (3)	C2F—C1F—C6F—N3F	175.6 (2)
O3C—N1C—C2C—C3C	−51.8 (4)	O6F—N3F—C6F—C5F	−10.1 (4)
O2C—N1C—C2C—C1C	−55.6 (4)	O7F—N3F—C6F—C5F	169.0 (3)
O3C—N1C—C2C—C1C	124.9 (3)	O6F—N3F—C6F—C1F	170.3 (3)
C1C—C2C—C3C—C4C	0.2 (5)	O7F—N3F—C6F—C1F	−10.6 (4)

### Hydrogen-bond geometry (Å, °)

**Table:**

Cg5 is the centroid of the C6B–C11B ring.

**Table:**

*D*—H···*A*	*D*—H	H···*A*	*D*···*A*	*D*—H···*A*
O2A—H2AD···O2B	0.84	1.80	2.638 (4)	180
N1A—H1AC···O1D	0.93	1.83	2.682 (3)	152
N1A—H1AC···O7D	0.93	2.63	3.301 (3)	129
N2A—H2AC···O1C	0.93	1.89	2.765 (3)	155
N2A—H2AC···O7C	0.93	2.46	2.990 (3)	116
O3B—H3BC···O3A	0.84	1.76	2.601 (4)	180
N1B—H1BC···O1E	0.93	1.85	2.678 (3)	147
N1B—H1BC···O7E	0.93	2.52	3.193 (3)	130
N2B—H2BC···O1F	0.93	1.91	2.764 (3)	153
N2B—H2BC···O7F	0.93	2.57	3.078 (4)	115
C19B—H19C···Cg5^i^	0.99	2.95	3.792 (4)	144

Symmetry codes: (i) *x*+1, *y*, *z*.

### Table 2 Cg···Cg π stacking interactions (Å)

Cg8 is the centroid of ring C1D–C6D and Cg9 is the centroid of the ring C1E–C6E.

**Table:**

	*Cg*X···*Cg*Y	CgX···Perp	CgY···Perp
Cg8···Cg9^i^	3.7419 (14)	3.2668 (9)	-3.3033 (9)

Symmetry code: (i) x, y,-1+z.
